# Informed Consent in Clinical Studies Involving Human Participants: Ethical Insights of Medical Researchers in Germany and Poland

**DOI:** 10.3389/fmed.2022.901059

**Published:** 2022-05-19

**Authors:** Cristian Timmermann, Marcin Orzechowski, Oxana Kosenko, Katarzyna Woniak, Florian Steger

**Affiliations:** ^1^Institute of the History, Philosophy and Ethics of Medicine, Ulm University, Ulm, Germany; ^2^Ethics of Medicine, Medical Faculty, University of Augsburg, Augsburg, Germany

**Keywords:** clinical trials, medical ethics, informed consent, Central and Eastern Europe, research ethics

## Abstract

**Background:**

The internationalization of clinical studies requires a shared understanding of the fundamental ethical values guiding clinical studies. It is important that these values are not only embraced at the legal level but also adopted by clinicians themselves during clinical studies.

**Objective:**

Our goal is to provide an insight on how clinicians in Germany and Poland perceive and identify the different ethical issues regarding informed consent in clinical studies.

**Methods:**

To gain an understanding of how clinicians view clinical studies in the countries they work in, we carried out semi-structured problem-centered interviews per telephone in Poland (*n* = 6) and Germany (*n* = 6). Our interviewees concentrated on three main topics: an appraisal of the normative framework, challenges in the information process and the protection of all participants in clinical studies.

**Results:**

Clinicians generally supported the normative framework, even though they considered it quite complex. In the two study countries, a widely noted dilemma in the information process was whether to overburden participants with extensive information or risking leaving out important facts. Clinicians were ready to exclude larger population groups from participating in clinical studies when the information process could not be carried out with standard procedures or when their inclusion was ethically sensitive.

**Conclusion:**

Clinicians need to gain a better understanding of the consequences of excluding larger population groups form participating in clinical studies. They should seek assistance in improving the information process for the inclusion of underrepresented groups in clinical studies.

## Introduction

Informed consent is a key principle in medical practice and clinical research involving human participants. It starts with the information transfer process between the clinical investigator and the research participant on the nature, purpose, risks, method and goals of the study, the rights and responsibilities of patients and their benefits, and the obligations of researchers. It continues with opportunities to clarify questions and assess the understanding of information, and culminates when consent is freely obtained or refused. The purpose of informed consent is patient information and seeking free and informed consent, or respecting dissent, thereby respecting autonomy and reducing harm (1, 2). An informed patient is also more likely to communicate researchers about possible factors affecting the clinical study, such as the intake of medications, sleep deprivation, or troubles adhering to prescribed diets (3).

It has been shown that despite shared acceptance of the norms listed in the Declaration of Helsinki, there are substantial differences on how Germany and Poland implement ethical norms related to patient protection in the different national laws (4). Cross-border clinical studies are becoming increasingly common, with a substantial increase in studies carried out in countries in of Central and Eastern Europe. The local availability of well-trained medical professionals and rapid patient recruitment make these countries especially attractive for international clinical studies (5, 6). When making use of the many advantages of carrying out studies in multiple sites, it is important that there is a common agreement on the implementation of widely recognized principles of research ethics, particularly on issues of informed consent and good scientific practice. The aim of our research is to provide an assessment on how the efforts to implement ethical norms in relation to informed consent in human subject research currently stand by analyzing the ethical insights of clinicians working in Germany and Poland.

There are a few factors that make a comparison of these two countries interesting from an ethics and policy development perspective. The collapse of socialists regimes in Eastern Germany and Poland marked a major change in the public perception of the role of the state and on how much citizens can trust the government to protect their interests, shifting values from strong welfare states toward Western free market ideals (7). Under the new social reality, the role of the public sector in securing the medical research needed to address public health issues is continuously shrinking, while the private sector's dominance is expanding (8). As strong governmental control and oversight declines, it is crucial that researchers are aware of their ethical responsibilities and institutional review boards are operating effectively to protect research participants. In the first two decades after the end of the Soviet era, we could find severe criticism on the slow progress in establishing independent institutional review boards and the implementations of international norms, such as the Declaration of Helsinki, in relation to informed consent in former socialist countries (9, 10). Furthermore, it is unclear how strong informal connections between clinicians, as central as they are in former socialist countries, allow sufficient enforcement of ethical norms in decentralized research systems (11, 12). A comparison of these two countries will provide us different perspectives on the implementation of research ethics norms in relation to informed consent in Germany and Poland from the practical experience of clinicians active in clinical research involving human participants.

## Materials and Methods

We conducted semi-structured problem-centered interviews to assess the different ethical perceptions among medical professionals working on clinical studies involving human participants. This type of interview allows to gain insight of the subjective perspectives of interviewees (13) and offers a certain flexibility by allowing to ask *ad-hoc* questions in order to clarify statements or to focus on particularly important issues. When necessary, follow-up questions to the main interview themes were asked (14).

**Figure 1:**
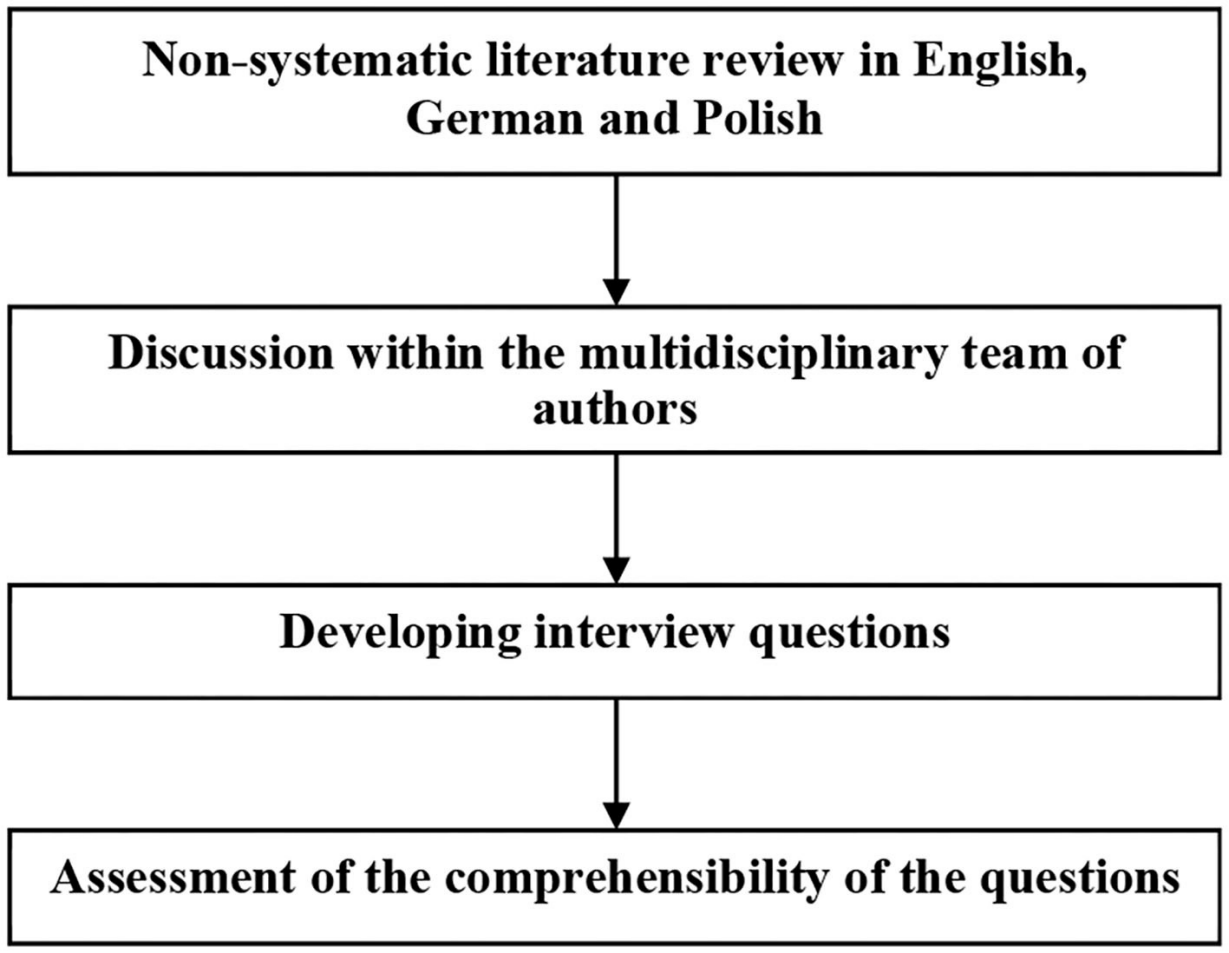
Schematic presentation of the process of developing the questionnaire.

We started our research with a non-systematic literature review through Google Scholar using the keywords “human subjects” OR “human participants” AND “clinical studies” OR “clinical trials” AND “ethics” to gain a general overview and included the search strings “Germany” OR “German” and “Poland” OR “Polish” to identify literature focusing on our case study countries. We repeated these literature searches using the equivalent search terms in German and Polish. This literature review, together with a discussion among the authors coming from different disciplines (medicine, medical ethics, medical history and political science), allowed us to develop a series of interview questions on the research topic. Figure 1 summarizes the process of developing the questionnaire.

Potential interviewees were identified through a web search and contacted via email. Here we followed purposive sampling to select participants who could provide important and diverse data (15). The criteria for participating in the interview was (i) to work in clinical studies on humans and (ii) to be a medical investigator or a research coordinator with medical training (Table 1). As the research did not seek to identify personal factors that influence ethical opinions, we did not collect further demographic information. We originally aimed at (*n* = 6) interviews in each study country: Germany and Poland. Candidates were approached with a short email with a brief introductory note to the project and a weblink presenting the main research team members. We sought informed consent on data use and explained the safeguards to guarantee anonymity. We carried out in each of the two countries *n* = 6 semi-structured interviews by telephone between March and April 2020 in the country's official language, i.e., Polish and German, by a female research team member with a PhD degree. The phone interviews were digitally recorded, transcribed and anonymized. Transcriptions of the Polish interviews were translated into English to be accessible for all authors. The interviews were analyzed following the method of structured qualitative content analysis as described in Mayring (14). We offer a thematic analysis following Braun and Clarke (16). First, we reduced the responses to their core elements, and then we manually coded, extracted and systematized these elements into main topics and subtopics. To avoid biases, the coding and analysis of the interviews was done by two researchers not involved in the interviews. These researchers worked independently on the coding and analysis. The results were later discussed and compared. We opted for a literal translation of the key terms in the interviews to facilitate transparency. After assessing the interview materials and questionnaires, the research team discussed the question of sufficient data saturation. Due to the nature of our qualitative study, we did not aim to provide a representative sample of medical professionals working on clinical studies.

**Table 1:** General information on interviewees.

	**Number of interviewees**	**Percentage of female interviewees**	**Median years of experience**
Germany	*n* = 6	50%	19
Poland	*n* = 6	50%	8

## Results

Our study results concentrate on three major topics: (i) appraisal of the country's normative framework, (ii) challenges and solutions to improve the informed consent process and (iii) special considerations for vulnerable groups. Table 2 provides an overview of the main topics in the responses.

**Table 2:** Main topics in the responses from the semi-structured interviews.

**Country**	**Main topics of the responses**
**Appraisal of the normative frameworks**	
Germany	Differences between state and federal regulations
	Multiple laws and regulations affect clinical studies
	European standardization was welcomed
Poland	Slow process in getting approval by bioethics committees
	Adequate safeguards for participants' autonomy
	Standardization of the normative framework
**Process and content of information**	
Germany	Clinicians pre-select information
	Documents need to be written in a simpler language
	Family doctors rarely inform about participating in clinical studies
Poland	Preference for extensive information content
	Documents need to be written in a simpler language
	Major prejudices exist against participating in clinical studies
**Vulnerable groups**	
Germany	Systematic exclusion of specific groups
	Language barriers hinder the inclusion of migrants
	Focus on compensation instead of therapeutic benefits
Poland	Inclusion of children whose parents work in different locations
	Family members exert pressure
	Migrants often avoid asking questions

### Appraisal of the Normative Frameworks

Although perceived as highly complex, half of the interviewees in Germany regarded the German framework as good. Nonetheless, it takes some considerable effort to learn how to implement and understand the normative framework in practice and quite some time to get familiar with the legal situation. Three interviewees explicitly stated that the German research environment was overregulated, two even claiming that it was excessively regulated. The shift toward European standardization was welcomed, even though some work is still needed to make international collaboration and comparisons easier. Standardization at a European and federal level would speed up research and make it easier to expand the study groups. One of the interviewed clinicians also pointed out that some standardization at the federal level on state [*Bundesländer*] laws and the requests and implementation of norms by ethics committees [*Ethikkommission*, institutional review boards] was desirable as well to facilitate multicenter studies. For instance, it was pointed out that it was tedious to learn “that, e.g., in Baden-Württemberg as a researcher I am theoretically allowed to exchange my data pseudo-anonymously with other researchers from other states according to the Hospital Act [Krankenhausgesetz], but not in Bavaria. In North Rhine[-Westphalia] again differently, in Brandenburg again not at all; that would be good, if there were simply uniform regulation, without that one must inform oneself always individually about it.” Standardization also reduces the risk that research partners who were part of the original group end up left out due to delays in getting at their local level the necessary ethics committee approval. As medical researchers in Germany hardly ever worked at the same University throughout their lives, learning new procedures for getting approval as they moved from one research center to another was seen as annoying and wasteful. One of the interviewees considered that the German legal framework was not well adjusted to realities and needs of medical research. None of the interviewees considered that having to inform patients about the European General Data Protection Regulation provides any added value for their protection, some even considered it as deviating the study participants attention from much more important issues.

For our comparative study, it was interesting to hear that one of the interviewees judged the German medical practice as promoting research subjects' autonomy by comparing the German situation to that of Eastern European countries, where based on anecdotal evidence, the interviewee judged physicians to be much more paternalistic.

There is a wide interest in participating in clinical studies in Poland, making the country an attractive site for clinical studies as recruitment is high. It was widely affirmed that standardizing legal norms would make it easier for sponsors to open facilities in Poland, which was seen as desirable. Here it is crucial that the normative framework is followed in each research site and loopholes are not abused or regulations misinterpreted. Slow processes and sometimes long waiting times for setting a date with bioethics committees [*komisja bioetyczna*, institutional review board] made Poland less attractive for carrying out research. Interviewees were content on how patient's autonomy was safeguarded, as ample opportunities to refuse participation and withdraw from studies were legally guaranteed and offered in clinical practice. A respondent was worried that many patients considered participating at clinical studies as the only way to get access to certain treatments. It is difficult to talk about free choice when there is scarce public health funding to secure access to non-experimental treatment alternatives. Despite this, the respondents raised no major complaints and had a positive attitude toward the Polish clinical studies environment. In Poland too, our respondents complained that informing about the details of the General Data Protection Regulation is a massive administrative burden that did not increase patient protection and was sometimes even counterproductive.

### Process and Content of Information

Among the interviewed clinicians in Germany, most were concerned about the abundant amount of information contained in informed consent documents. Providing large amounts of information was not seen as supportive for informed consent, but rather as a risk, as patients may oversee critical information surrounded by other information they may deem as less relevant. The clinicians frequently pointed out that much of the material that was handed out was too long and too difficult to comprehend. While reading capacity of an average school level was targeted, many materials were assessed by linguists as University entrance level, or even requiring a natural science degree. An interviewee even suggested to take a much lower reading level “One would actually have to take tenth grade level, compulsory education in Germany is until 10th grade.” An additional problem with many international studies, was that the translation of the information materials was not adequate, and often more difficult to understand.

Two interviewees perceived the reflection period, often lasting 24 h or more, between the information processes and actual consent as a very good instrument to protect patient autonomy. Patients however rarely used this time as an opportunity to study the information materials carefully at home. The interviewees did not consider that the family of the participants played a significant role in the decision to participate in the studies and clearly stated that participation is an individual choice. Only one respondent witnessed that the opinion of the patient's partner did play a significant role in the decision. One of the respondents reported that their clinic specifically scheduled a longer physician-assisted information process for cases where information materials extended over several pages. In general, the interviewees saw the role of ethics committees in protecting patient autonomy as very positive. It needs to be noted, that the remarks of many respondents hinted that they were outsourcing the ethical decisions to the ethics committee, relying for both ethical and legal reasons primarily on the committee's judgement.

Two interviewees were worried that family doctors [*Hausärzte*, general practitioners] generally did not inform patients about the possibility to participate in clinical studies, some even showed a negative disposition toward their patients participating in such studies. Many patients actively sought to participate in clinical studies after reading advertisements. For cancer patients, family members actively searched for clinical studies as an option to seek new medical treatments. The interviewees saw that the public had a negative view on clinical studies and considered that prejudices and misinformation deprived them of an important opportunity to find a possible treatment for their condition.

In Poland a physician complained that consent forms were insufficiently patient-centered and rather conceived to protect the clinical studies sponsor. The introduction of the General Data Protection Regulation worsened this situation, while hardly offering additional protection to patients. Documents need to be written in a simpler language. Here too, one respondent stated that “if we give too much information, the most important information is blurred.” Nonetheless, it was recognized that clinical studies sponsors had a strong incentive to provide well-written and properly translated information forms. Yet, here also one of the respondents complained about the quality of the translations and the failure to adapt the information materials to national context. Many patients actively sought to participate in clinical studies, as they saw such studies as an opportunity to access treatment options not covered by health insurance. By looking for such studies on the internet, these patients already arrived with some knowledge about the nature of clinical studies in general and the studied clinical intervention. In many cases, participants took home the information materials to discuss their involvement in the study with relatives or their family doctor.

Physicians needed to clear out deeply anchored prejudices and biases to ensure that patients really considered participation in clinical research as an option for receiving a state-of-the-art treatment and examination. While for many people participating in clinical studies was seen as a good way to access novel treatment options, in some cases patients did associate these studies quite negatively with being a “guinea pig.” Some patients thought that “companies want to take advantage of Polish patients”, without being fully aware that clinical studies are subject to strict regulations and patient protection measures. Such stereotypes were particularly prevalent among less educated patients and were often an impediment to participate in clinical studies, even when it was of their clear advantage. A negative inclination toward clinical studies was particularly strong among people who knew that medical investigators were well remunerated. While patients were informed that they could retract from a study without having to give any reasons, medical investigators were often requested to provide an explanation to sponsors on why participants left, which made it difficult to refrain asking the patients who wanted to leave further questions. A physician pointed out that the information process could be significantly improved if physician could allocate more time in adequately informing the patient. Lastly, the patients need to understand that they not only have rights during clinical studies, but also certain responsibilities.

### Vulnerable Groups

In Germany, one of the interviewees was very clear in labeling the wide exclusion of pregnant women and breast-feeding mothers as a major ethical problem, as such practice fails to identify risks and benefits of medicines for women and their offspring in such stages thereby leaving them without a proven and safe medical treatment. Many clinicians did not have any experience including pregnant study participants. Attempts to protect patient autonomy can lead to the injustice of not examining the effectiveness and safety of medical procedures for the specific profile of whole groups of people.

A clinician stated that their institution did not carry out studies on people who could not read the information materials in German. It was rare that patients who had a migrant background took part in the studies. Patients from low socio-economic groups concentrated their attention to the amount they will receive as an allowance [*Aufwandsentschädigung*, compensation for expenses], while wealthier patients were more interested in the therapeutic effect of the study. Children were difficult to include in the studies, but their presence was deemed necessary and the lack of data on how minors react to treatment was seen as a medical problem. It was rare that parents and children disagreed about participating in the studies, although minors are becoming increasingly critical.

In Poland, one of the interviewed clinicians mentioned that in their facility they did not do studies that included children. Due to the large number of separated parents and people who work for extensive periods abroad, safeguards requiring the consent of both parents for minors were often a major impediment for facilitating participation.

Family members may exert significant pressure to participate in a study or withdraw from it. In some cases, family members are directly involved in seeking new information from the internet and asking the study organizers questions. In this regard, patients with cancer were much more open to participate in clinical studies.

People with a low level of education were perceived as more vulnerable to paternalistic attitudes, requiring physicians to be particularly sensitive during the information process. People from rural areas and more traditional cultural backgrounds, particularly from Eastern Poland and migrants from Ukraine and other Eastern countries, often avoided asking physicians questions. Migrants from Ukraine frequently associated a failure to consent to studies with a loss of entitlement to any type of medical treatment. For instance, a clinician noted that “they assume that you should sign [the consent form], otherwise you won't get treatment. And that's it.” For people in rural areas and people with very limited financial resources participation in clinical studies was seen often as the only way to access treatment options. One clinician reported that when a communication problem became apparent at the beginning of patients' participation in a clinical study, their participation was terminated. Others were more open to adapt information to make it more accessible for people with poor Polish language skills.

## Discussion

We proceed by discussing central ethical issues based on the responses of our interviewees: (i) concerns related to the information content and strategies used to improve the information process, (ii) specific challenges to respect autonomy and promote the well-being of vulnerable groups, and (iii) remarks on the ethical reasoning of clinicians.

### Information Content

A central worry in relation to patient information was the amount and scope of information that was provided to patients. Here our respondents focused on the challenges in informing patients about (i) the experimental nature of the studies, (ii) the risks of the studies, (iii) the benefits of participation, (iv) the rights of patients, particularly the right to quit without providing a justification, and (v) the data protection measures. In view of internationally agreed standards (17), it was interesting to see that our respondents did not disclose any information on communicating the overall study design and methods to patients, other than the factors directly affecting the participant being informed. Such omission may result in patients not disclosing important information that may affect research results and paying insufficient attention in following the requests from the research team, such as adhering to diets, alcohol and tobacco restrictions, or reporting the use of certain medicines (3). Patients need to become aware, that not only passive participation is requested, but also a certain commitment to avoid and report anything that may distort the study results. Only one interviewee explicitly mentioned that patients not only have rights, but also acquire duties when consenting to participate in a study. It is understandable that clinicians may be hesitant to provide information on the overall study design. There is already a widely acknowledged problem of overburdening patients with information, something that is also recognized in the normative frameworks of the study countries (4). Informing patients about possible rare side effects may trigger certain effects that can harm them and distort studies, placing physicians in a difficult ethical dilemma concerning the nocebo effect (18). However, leaving out information may omit crucial information. For instance, a survey conducted in Poland revealed that 14% of healthcare professional seldom inform patients about possible negative effects of an experimental therapy (19). Moreover, reducing the information burden for patients comes with a selection bias that may be tainted toward the interests of the researchers. Several of the interviewed clinicians recognized this problem and systematically schedule more time for a physician consultation as information material gets longer and offered supporting information that can be accessed over the internet beforehand.

The research team should not be left alone in improving patient information. As medical research is a public good that benefits society as a whole (20), governments need to implement educational campaigns about the importance of participating in clinical studies and explain the many potential benefits for individuals and society, and the safeguards that are implemented to reduce risks and the compensation for eventual harms. Essential is also to clearly communicate to participants, whether the study can be therapeutically beneficial for them (21) and how risks can be minimized. Often participants are convinced that participation in a clinical study will improve their health—a so-called “therapeutic misconception” (22, 23). Such efforts will help researchers to recruit participants and destigmatizes participation. The recurrent reference to not wanting to be a “guinea pig,” particularly in Poland, shows that stigmas and aversions to clinical studies are widely present (24). The consequences of decades-long antagonism between Western and socialists countries still reflect in mistrust among certain population groups toward international research collaborations (25).

### Respect for Autonomy and Beneficence: Vulnerable Research Subjects

Clinicians in both study countries had few problems excluding wider population groups from clinical studies. Views supporting a right to participate in clinical studies, or even that people have a duty to contribute to clinical research (20), were rarely held. Such blanket exclusions go against both the autonomy and beneficence of patients (26). For instance, in Germany, the interviewed clinicians generally excluded participants who were not proficient in German, reporting no adaptations to facilitate participation in cases where the information process could have been carried out in another shared language, like English. Although there is not sufficient data on migration status of participants in clinicals studies in Germany, other authors have also reported that rarely institutional review boards receive documents in other languages, for instance Turkish, for approval (27). The inclusion of migrants in clinical studies requires from clinicians to be able to distinguish between the different types of literacies, i.e., linguistic, scientific and technological, adapt the information process accordingly and assess whether communication barriers can indeed be lifted. Furthermore, researchers need to be aware of general sociocultural circumstances, such as the fasting during Ramadan of Muslim migrants (28). Distrust in the medical system can also be a factor limiting participation of migrants and minorities and therefore needs to be actively confronted (29).

The inclusion of children in the informed consent process remains a major problem, as it remains necessary to assess the therapeutic benefits and risks of clinical interventions for this age group (30). The information process concerning children needs to be improved (31). Polish researchers have suggested to take a more dynamic approach in adapting the age limit for children's assent to the nature of the study and the child's cognitive capacity (32). Empirical studies have shown that the majority of minors age 12 and above are capable of understanding the basic elements of informed consent forms (23). Furthermore, due to the large number of divorced parents and cases where one of the parents works abroad, regulations need to make it feasible for children whose parents do not share a common household to participate in clinical studies.

Another controversial aspect was the general reluctance to include pregnant women or even institutional policies that strictly forbid such inclusion (33, 34). Such policies have been identified as disrespectful toward women's autonomy (35). It is imperative that clinicians recognize how exclusionary policies not only deprive patients from access to important treatment options (36), but also fail to recognize patients that are labeled as “vulnerable” as autonomous agents that are capable of making difficult choices when properly informed.

### Observations on the Ethical Reasoning of Clinicians

Physicians occasionally judged the ethical acceptance of their own clinical practices as superior by claiming that in other countries paternalism and failure to obtain informed consent were widespread—often without hard evidence. Such approach may lead to being less strict about one's own standards, when one is convinced to be the only one that sticks at the established rules. Instead, physicians should aim at meeting ethical ideals without pointing to possible non-compliers to deviate attention from their own practices and avoid self-criticism.

Another worrying aspect, is that many medical investigators delegated much of the ethical decision-making to institutional review boards (37). While ethics committees may have more expertise on ethical matters than biomedical researchers, they do not have access to as much information as the research team, nor can they witness the emotional expressions and reactions of patients during the clinical studies. To safeguard participants and avoid carrying out studies without social value, the research team needs to keep its own ethical judgement criteria and be open to express doubts and consult unforeseen matters with institutional review boards even after having received their approval. If ethics committees are asked in the future to assume more than an advisory role, they will need new tools and resources (38).

Diversity within medical research teams revealed itself as a very positive aspect for patient participation and for removing knowledge gaps. When researchers reflect from their own vulnerable position or circumstances, they can avoid situations where they would lean toward paternalism. A female researcher for instance pointed out the importance of including pregnant study participants, as she herself would want to know that medicines have been adequately verified for pregnant women when she herself was in that situation. Most interviewees were well familiar with the realities in rural contexts—particularly in Poland—and other cities within the country, suggesting significant mobility among medical professionals at the national level. Our study design did not allow us to assess the perspectives from physicians with migrant background, something that needs further studies.

### Limitations

In our qualitative study we identified two participation biases. Many interviewees specifically mentioned that they wanted to participate as they themselves were researchers. Others were unsatisfied with the current system and participated in the hope to make a small contribution to change the system.

Our study design aimed at analyzing ethical insights of clinicians working with human subjects in Germany and Poland using qualitative research methods. Therefore, it cannot be representative for the opinion of all clinicians working in these countries. Despite informing interviewees that we will anonymize their responses, it was unlikely that interviewees reveal obvious malpractices. Further work needs to study the impressions of study participants in these two countries to identify possible weak spots, preferences, expected level of involvement, and assess the effectiveness of patient information strategies (39).

## Conclusion

As we could conclude from our survey, there are both common and specific problems in the compared countries. Common problems include an imperfect legislative framework to guide ethical decision-making, the need for clearer regulation of vulnerable population groups, and often difficulties in communicating information about clinical studies to the patient.

Particularly for Germany, clinicians found it quite burdensome that there were regulatory differences between the different states of the federal republic. Polish clinicians complained that regulations concerning the inclusion of children were not drafted in consideration to the new realities in which children are currently raised.

In the two countries we could observe a general tendency to avoid including participants from groups that are particularly difficult to inform or whose inclusion may be perceived as ethically controversial. The interviewed clinicians were very cautious about not risking to harm participants. We observed that most of the clinicians gave priority to the ethical principle of non-maleficence, interpreted narrowly in the sense of not doing anything that may harm participants, instead of broadly, which would also consider exclusion as a form of harm. Indeed, the systematic exclusion of people from clinical studies was rarely seen as something negative. Only a few clinicians pointed out that exclusions hinder people to benefit from participating in clinical studies (principle of beneficence). It was rare to hear about references to respecting autonomy in the decision over participation or even to refer to exclusion as a form of injustice. Even though the principle of justice is a fundamental principle of biomedical ethics, it was surprising to observe that the interviewees did not perceive large exclusions as a form of injustice. Further studies are needed to assess, whether physicians in the two countries have indeed an adequate understanding of this ethical principle and if this needs to be improved in medical ethics training. It is also important to note that both countries need to work toward adjusting their norms and practices to the present-day internationalization of the job market. Germany needs to work toward lifting language barriers to expand participation within its migrant population and Poland will have to offer alternative forms of giving parental consent for children whose parents work in different cities or even countries. Identifying difficulties in obtaining informed consent should not ethically legitimize the blanket exclusion of whole groups, but should invite clinicians to seek help in designing more accessible information materials and work with other experts to improve the information process.

Regarding the amount of information offered during the informed consent process, there are arguments in favor and against giving participants extensive information or allowing clinicians to preselect the information shared with participants. The former was common among Polish interviewees, the latter among the interviewed German clinicians. There is no consensus in medical ethics on which approach should be followed, as the scholarship on possible biases when selecting information is particularly active. Further studies are needed to clarify this question by taking special consideration to the different forms and levels of literacy.

Patient information process remains challenging for all groups. Providing information on websites so that potential participants can gather preliminary information in advance was perceived by our interviewed clinicians as a successful strategy to decrease information overload during the informed consent process while at the same time increasing public awareness on the safeguards implemented to reduce risks and the potential benefits of participation.

## Data Availability Statement

The data presented in this study are available on request from the corresponding author.

## Ethics Statement

The study was reviewed and approved by the Ethikkommission der Universität Ulm. Written informed consent for participation was not required for this study in accordance with the national legislation and the institutional requirements.

## Author Contributions

CT, MO, KW, and FS: conceptualization. CT, MO, and OK: data analysis. CT: writing—original draft preparation. MO: funding acquisition. CT, MO, OK, KW, and FS: writing—review and editing. All authors contributed to the article and approved the submitted version.

## Funding

The research was supported by the Graduate and Professional Training Center Ulm University (ProTrainU) within the framework of the start-up research financing program for young scientists.

## Conflict of Interest

The authors declare that the research was conducted in the absence of any commercial or financial relationships that could be construed as a potential conflict of interest.

## Publisher's Note

All claims expressed in this article are solely those of the authors and do not necessarily represent those of their affiliated organizations, or those of the publisher, the editors and the reviewers. Any product that may be evaluated in this article, or claim that may be made by its manufacturer, is not guaranteed or endorsed by the publisher.

## Appendix

Questionnaire guiding the interviews.

1. Legal and ethical aspects of research involving human participants

- How do you evaluate the legal situation of research on human beings in your country?
- What fundamental ethical conflicts can you identify in the field of research involving human participants?

2. Self-determination of patients

- How do you evaluate the legal regulations on patient self-determination?
- Where do you encounter difficulties in your everyday practice in preserving patient self-determination?

3. Patient information

- What information do you provide to patients during the informed consent process?
- How do you deal with cultural and social differences in the understanding of clinical trials (e.g. the understanding of risks)?

4. Protection of person-related data

- What are your thoughts on data protection and the new data protection regulation?
- What ethical conflicts do you see in the processing of person-related data in the field of clinical studies?

5. Access to clinical studies

- Access to clinical studies is regulated differently in European countries. What legal restrictions are necessary in your view? Which groups need special protection?
- What ethical concerns do you have about study participants who are incapable of giving consent?

6. Need for improvement

- In your opinion, which legal and ethical aspects are neglected in research involving human participants?
